# Are the Anterior and Mid-Cingulate Cortices Distinct in Rodents?

**DOI:** 10.3389/fnana.2022.914359

**Published:** 2022-06-02

**Authors:** Jose Francis-Oliveira, Owen Leitzel, Minae Niwa

**Affiliations:** ^1^Department of Psychiatry and Behavioral Neurobiology, Heersink School of Medicine, The University of Alabama at Birmingham, Birmingham, AL, United States; ^2^Department of Neurobiology, Heersink School of Medicine, The University of Alabama at Birmingham, Birmingham, AL, United States; ^3^Department of Biomedical Engineering, School of Engineering, The University of Alabama at Birmingham, Birmingham, AL, United States

**Keywords:** anterior cingulate cortex, mid-cingulate cortex, prelimbic cortex, layer V, layer VI

## Abstract

The prefrontal cortex (PFC) is involved in cognitive control, emotional regulation, and motivation. In this Perspective article, we discuss the nomenclature of the subdivisions of the medial prefrontal cortex (mPFC), since the anatomical definitions of the PFC subregions have been confusing. Although the mid-cingulate cortex (MCC) and anterior cingulate cortex (ACC) have distinct features in humans and non-human primates, it is unclear whether these regions serve different functions in rodents. Accurate mapping of the cingulate cortex in rodents is important to allow comparisons between species. A proposed change in the nomenclature of the rodent cingulate cortex to anterior cingulate cortex (aCg) and mid-cingulate cortex (mCg) is presented based on our data. We show evidence for distinct cortico-cortical projections from the aCg and mCg to the PrL. The aCg→PrL neurons were abundant in layer VI, while the mCg→PrL neurons were mainly distributed in layer V. In addition, a sex difference was detected in the aCg, with males having a higher proportion of layer V neurons projecting to the PrL than females. Based on this laminar distribution and considering that layer V and VI send efferent projections to different brain areas such as the brain stem, amygdala, and thalamus, we propose that aCg and mCg need to be considered separate entities for future rodent studies. This new definition will put into perspective the role of rodent cingulate cortex in diverse aspects of cognition and facilitate interspecies comparisons in cingulate cortex research.

## Introduction

In humans and rodents, the prefrontal cortex (PFC) is regarded as a hub brain region that controls cognition and affection (Brunson et al., 2003; Robbins, 2005; Arnsten, 2009; Dajani and Uddin, 2015; Sandi and Haller, 2015; Uddin et al., 2019). Research on the PFC has been confounded by confusing anatomical and functional definitions of its subdivisions, especially in rodents (Laubach et al., 2018; van Heukelum et al., 2020). In recent decades, more precise anatomical distinctions have been made in human and primate studies, but homologies between rodent and human/primate brains remain unclear (van Heukelum et al., 2020). Therefore, the terminology of the cingulate cortex in rodents is confusing and in need of revision.

Several studies have been conducted on the anatomy and neurophysiology of the cingulate cortex (Ahmed et al., 1995; Vogt, 2016). Studies in rodents have shown that inputs from ipsilateral PFC areas reach the cingulate cortex (Ahmed et al., 1995). This connectivity may be reciprocal (Hoover and Vertes, 2007; Qadir et al., 2018). The literature on this topic in rodents is still new, and little is known about the laminar distribution of these projecting neurons in rodent cortico-cortical networks. Moreover, it is not clear if sex or age differences exist in such circuits. Are there differences in the laminar distribution between neuronal projections in cingulate cortex? Are there any sex differences in these circuits? Answers to these questions are needed to fill the gaps in our knowledge about the laminar structure of rodent cortico-cortical circuits, since rodents are used in many studies that aim to investigate how neuronal circuits regulate cognition. Here we present a new perspective on the neuroanatomy of the cingulate cortex in rodents.

## A Revision of the Anatomical Definitions for the Cingulate Cortex

**FIGURE 1:**
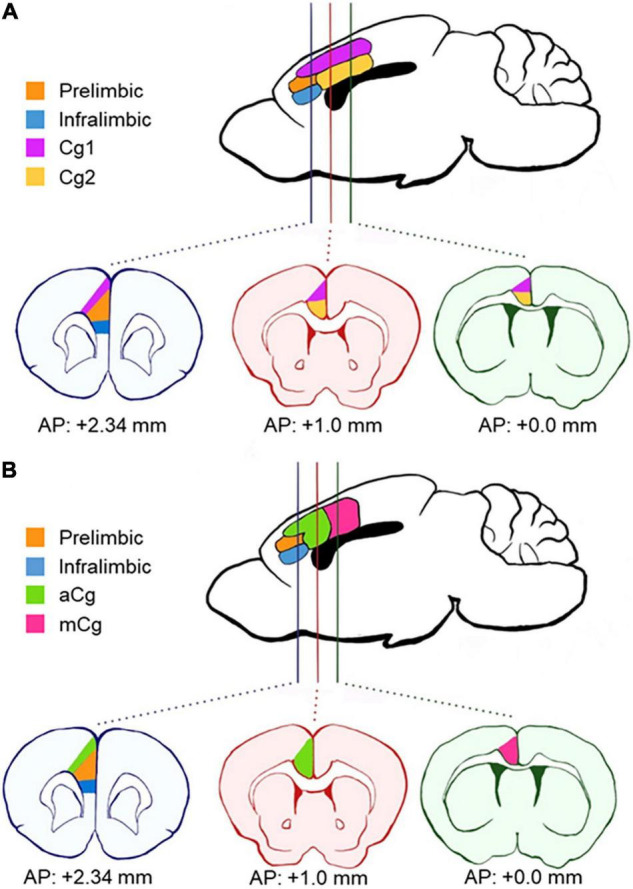
Schematics of anatomical divisions in the prefrontal cortex. **(A)** Areas according to Paxino’s Mouse Brain Atlas (Franklin and Paxinos, 2013). The cingulate cortex is divided horizontally into two distinct regions across the coronal plane (Cg1 and Cg2). **(B)** Areas according to our definition, based on van Heukelum et al. (2020). Here, the cingulate cortex is divided into the anterior cingulate cortex (aCg) and mid-cingulate cortex (mCg) in the coronal plane. aCg and mCg both encompass regions Cg1 and Cg2 as defined by Paxino’s Atlas. AP, antero-posterior coordinates from bregma.

The medial portion of the PFC is defined as the “medial prefrontal cortex (mPFC),” which in rodents is further subdivided into the infralimbic, prelimbic (PrL), and Cg1/Cg2 regions (Franklin and Paxinos, 2013; Figure 1A). In human and primate studies, these same regions are often referred to as the “anterior cingulate cortex (ACC).” Experiments in humans failed to demonstrate a homogenous activation of ACC under a variety of contexts (Vogt et al., 1992). In humans and primates, the ACC has been recognized as a region composed of distinct subregions, Brodmann areas (BA) 32, 24a, b, c, and 25, based on cytoarchitectural differences. These regions are mapped onto three general regions: ventral ACC (BA 25), rostral ACC (BA 32), and dorsal ACC (BA 24b, c), with different connectivity (Shackman et al., 2011; Vogt and Paxinos, 2014). In particular, the mid-cingulate cortex (MCC) is also referred to as the dorsal ACC in humans and primates (Shackman et al., 2011). However, it is still unclear how these regions differ functionally. Due to the unknown existence of the MCC homolog in rodents, there is a lack of research studies on the specific cingulate cortex circuits *in vivo*, which are usually performed in rodents. Mapping the rodent cingulate region in a homologous manner to primates and humans has been an important outstanding question in the field. Therefore, this Perspective article presents a unique and important viewpoint using mouse tracer data to clarify these issues.

The primate mPFC is often referred to as ACC in rodents (Laubach et al., 2018; van Heukelum et al., 2020), which explains why the terms “mPFC” and “ACC” have been used interchangeably in rodent research, causing confusion in the terminology. The primate mPFC refers to BA 32, 25, and 24 of ACC, and some PFC regions that are not present in rodents (BA 9, 10, 11, and 14), which suggests that rodent mPFC is homologous to the primate ACC region (Laubach et al., 2018). However, many rodent studies target different prefrontal regions and refer to all of them simply as “ACC.” Also, unlike the anatomical definitions for primates and humans, the “MCC” for rodents is still undefined. To clarify the subregions of the mPFC in rodents (or the ACC in human and primate research), a new division in the coronal plane has been proposed in the last decade (Shackman et al., 2011; van Heukelum et al., 2020). This proposal accounts for differences in cytoarchitecture and connectivity (Vogt, 2016). The proposed division defines the PrL and infralimbic cortex within the mPFC in rodents and distinguishes the cingulate cortex regions along the rostro-caudal rather than the dorso-ventral plane (Figure 1B). These two cingulate cortex regions encompass both the previous regions, Cg1 and Cg2, in each new division. The new regions are defined as the anterior cingulate cortex (aCg) and the mid-cingulate cortex (mCg) in rodents (Figure 1B). The aCg begins around the antero-posterior coordinates (AP) + 1.0 mm from bregma and lateral to the PrL (AP + 2.34 mm from bregma). The mCg starts around AP + 0.0 mm from bregma (Vogt, 2016; van Heukelum et al., 2020).

This new definition is consistent with neuronal connectivity. The aCg defined in this way shows strong connections to autonomic nuclei, amygdala, and hypothalamus, while the mCg had stronger connections to other cortical areas such as the parietal and retrosplenial cortex (Hoover and Vertes, 2007; Fillinger et al., 2017). In contrast, the PrL receives projections from nearby cortical areas involved in attention control and decision making (Qadir et al., 2018), suggesting an important distinction between the cingulate cortex and PrL. Defined in this manner, the rodent aCg correlates to BA 24, 25, and 32, and the mCg correlates to BA 24′ (Fillinger et al., 2017; van Heukelum et al., 2020).

## Laminar Distribution of Cingulate Cortex Projections to Prelimbic Cortex

Another feature that distinguishes the aCg from the mCg is the laminar distribution of its projections to the PrL in rodents. The cingulate cortex consists of neurons distributed in layers which organize the computation and integration of many different inputs (Larkum et al., 2018). In general, subregions of the cingulate cortex contain layers I, II/III, V, and VI (layer IV is absent). In layer I, interneurons are sparsely distributed, and most of this layer is composed of axonal projections from other brain regions (Ibrahim et al., 2020). Neurons in layers II/III are mostly pyramidal and receive inputs from layer I. Layers II/III neurons output to pyramidal neurons in layers V and VI, which compute the information flowing through the cortical network before sending efferent projections to thalamus, brain stem nuclei, and other cortical regions (JLi et al., 2009; Fillinger et al., 2017). Neurons in different layers have different electrophysiological properties (Correa-Junior et al., 2020). Layers V and VI neurons have distinct molecular and cellular properties and functions (Baker et al., 2018). However, studies on PFC areas, such as the cingulate cortex, usually combines cortical layers V and VI together as “deep layers” and treats them as a single entity (Baker et al., 2018). Circuits containing neurons in layer V will be distinct from those arising from layer VI, but the specific roles of layers V and VI remain elusive.

Characterization of neurons by their location in a certain cortical layer may be misleading due to the complex dendritic interactions in cortical circuits (Spratling, 2002; Stuart and Spruston, 2015). Nonetheless, this classification is a very important step toward a better understanding of the roles of specific neurons. For instance, grouping neurons in cortical layers has allowed the identification of feedforward and feedback inhibition loops across layers in the sensory cortex (de Kock et al., 2012; Bojak et al., 2015). In the aCg, electrophysiological studies have demonstrated connections between layers I and II/III (JLi et al., 2009; Larkum et al., 2018). Despite many studies being conducted to understand the integration of information in cortical networks (de Kock et al., 2012; Stuart and Spruston, 2015; Larkum et al., 2018), the field remains focused on whole brain regions. The roles of specific cortical layers in behavior and other neuronal functions have not been well explored, until more recently that such investigations can be done with new techniques including *in vivo* activity imaging in rodents (Gulati et al., 2017) and *in vivo* laminar recordings in monkeys (Chandrasekaran et al., 2017).

**FIGURE 2:**
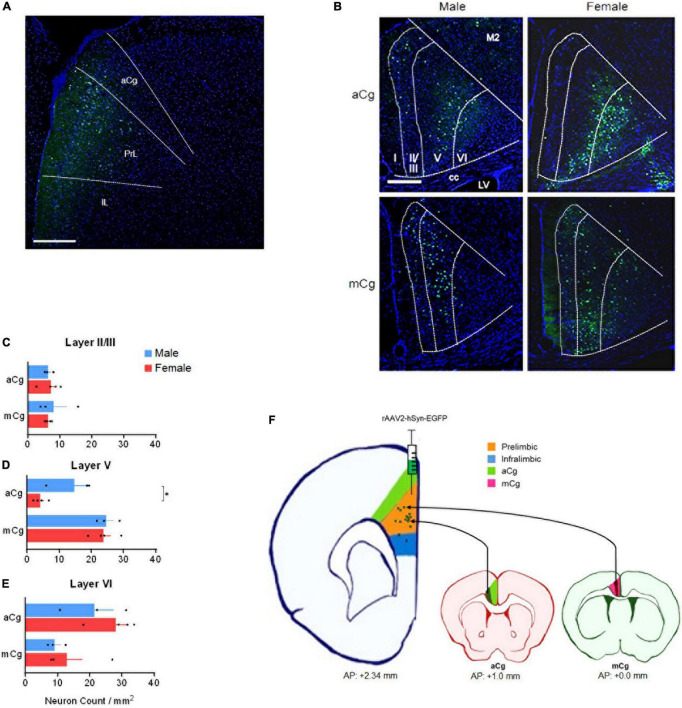
Laminar distribution of aCg/mCg→PrL neurons, in male and female mice. **(A)** Representative image identifying the injection sites in the prelimbic cortex (antero-posterior from bregma, +2.34 mm; mediolateral from bregma, +0.35 mm; dorso-ventral from dura, –1.95 mm) (Franklin and Paxinos, 2013) of an adult C57BL/6J mouse. The final titer of the virus was 7 × 10^12^ viral particles per ml. aCg, anterior cingulate cortex; PrL, prelimbic cortex; IL, infralimbic cortex. Scale bar = 500 μm. **(B)** Representative images of the aCg and mCg from C57BL/6J male and female mice. EGFP-labeled neurons in aCg and mCg were observed ipsilateral to the virus injection site of PrL. A smaller proportion of neuronal labeling on the contralateral side was also observed. DAPI was used as counter-staining. M2, motor cortex; cc, corpus callosum; LV, lateral ventricle. Scale bar = 500 μm. **(C)** Counting of aCg/mCg→PrL projections located in layers II/III. Two-way ANOVA for interaction: *F*_3,10_ = 0.036, *p* = 0.852; main effect for brain area: *F*_1,10_ = 0.038, *p* = 0.850; main effect for sex: *F*_3,10_ = 0.026, *p* = 0.874. **(D)** Counting of aCg/mCg→PrL projections located in layer V. Two-way ANOVA for interaction: *F*_3,10_ = 3.804, *p* = 0.080; main effect for brain area: *F*_1,10_ = 37.114, *p* < 0.001; main effect for sex: *F*_1,10_ = 5.511, *p* = 0.041. Bonferroni: aCg Female × Male, *p* = 0.012. **(E)** Counting of aCg/mCg→PrL projections located in layer VI. Two-way ANOVA for interaction: *F*_3,10_ = 0.126, *p* = 0.730; main effect for brain area: *F*_1,10_ = 9.932, *p* = 0.010; main effect for sex: *F*_1,10_ = 1.398, *p* = 0.264. *n* = 6 for males (*n* = 3 for aCg, *n* = 3 for mCg), *n* = 8 for females (*n* = 4 for aCg, *n* = 4 for mCg). All data are presented as mean ± SEM, each dot represent the value of one individual animal. *p < 0.05. **(F)** Injection sites in the PrL (green dots in the orange shade). Two animals were excluded from the analysis because they were injected into the infralimbic cortex (x in the blue shade). Projections from aCg originated mostly from layer VI (dark green shade), and those from mCg originated mainly from layer V (dark red shade).

Injecting retrograde viruses in the PrL of adult mice (10–15 weeks of age) from both sexes (rAAV2-hSyn-EGFP, a gift from Bryan Roth, Addgene viral prep # 50465-AAV2, 200 nL, unilateral) allowed us to see that the laminar distribution of aCg/mCg neurons projecting to the PrL is contrastingly different (Figure 2). We allowed 2 weeks for the viral expression, and perfused mice with phosphate buffered saline followed by 4% paraformaldehyde. Slices containing the PrL were obtained to validate the injection sites (Figures 2A,F) and aCg and mCg slices were obtained for analysis under a fluorescence microscope. The aCg→PrL neurons were mainly distributed in layer VI, while the mCg→PrL neurons were predominantly located in layer V (Figures 2D,E). Using the common classification of Cg1/Cg2 areas for mice (Franklin and Paxinos, 2013), there was no difference in neuronal labeling among these cingulate areas. This observation is consistent with the new nomenclature for the cingulate cortex in rodents proposed in this Perspective article, indicating that in rodents, aCg and mCg are two distinct brain regions rather than a single entity.

## Are There Different Functional Roles for aCg and mCg?

Projections from aCg/mCg to the PrL in mice arise primarily from layers V to VI, as observed in Figure 2. Labeling was also present in layer II/III, but no labeling was observed in layer I. aCg and mCg neurons may control distinct behavioral aspects based on the laminar distribution of their projections to PrL and other targets in the brain. In the aCg, neurons are involved in controlling stress and emotional responses through their layer VI output (JLi et al., 2009; Fillinger et al., 2017), which may include the PrL as a target. This suggests that integration of information occurs through a cortico-cortical loop, and the resulting information is sent to amygdala and brain stem regions via efferent projections from the PrL (Etkin et al., 2011). In contrast, given that the mCg projects to other cortical regions, the output information from mCg layer V neurons to PrL may be involved in cognitive control, fear, and aggression (van Heukelum et al., 2019b). As both aCg and mCg projects to PrL, the cingulate cortex as a whole may have a complex intracortical network with cortico-cortical loops, which may play an important role in integrating information in these regions. However, since the mCg is often undefined or misrecognized as the ACC or other mPFC regions in rodent studies, comparing functional differences between aCg and mCg has been difficult. Taken together, our data support the anatomical division of the mouse cingulate cortex into aCg and mCg with distinct roles for these two subdivisions based on different laminar distributions.

To integrate many distinct stimuli and coordinate behavioral responses, communication between the PrL and other cortical regions may be necessary. The PrL has different neuronal characteristics and projections, suggesting heterogeneity of neurons that may be implicated in different functions. There is evidence for such heterogeneity in the PrL regarding the monoaminergic receptor profiles (Santana and Artigas, 2017), the functional connectivity of PrL layers II/III and V neurons to amygdala (Avesar et al., 2018), and subpopulations in PrL layer V projecting to different regions (Santana and Artigas, 2017; Avesar et al., 2018; Cruz et al., 2021). The laminar distribution of aCg/mCg→PrL projections may also correspond to a laminar distribution of their PrL targets. Moreover, PrL neurons may have distinct molecular profiles correlated to receiving projections from aCg/mCg.

Taken together, we speculate that aCg and mCg may perform different roles in behavioral modulation. The current terminology treats both rodent aCg and mCg as a single entity, which may prevent us from demonstrating differences or effects in functions regulated by the cingulate cortex. To our knowledge, although there are a few behavioral studies comparing aCg and mCg in rodents, cognitive aspects of behavior have not been evaluated in detail (van Heukelum et al., 2019a,b; Liu and Lai, 2021). Thus, while there is strong evidence for functional differences between the ACC and MCC in cognition in primates, this exploration is lacking in rodents. In the future, the functional aspects of the circuits linked to aCg/mCg and PrL in rodents need be investigated using this new anatomical definition for the cingulate cortex.

## Are There Sex Differences in Cingulate Cortex Projections?

Developmentally programmed sex differences occur in the brain, where a few sex dimorphisms have been demonstrated in both rodents (AGordon et al., 1978) and humans (Witelson et al., 1995). Sex differences have been subject to controversies in behavioral neuroscience and have often been overlooked in studies because female animals have not been used or differences are too subtle to be detected (McEwen and Milner, 2017). Understanding such differences in neuronal circuits may be the key to resolving the differences in susceptibility to behavioral changes between sexes.

In our data, male mice showed a higher density of aCg→PrL neurons in layer V, compared to females (Figure 2D). Although many behavioral changes in humans are strongly influenced by sex, the neurobiological mechanisms for this fact remain unclear. Differences in the aCg/mCg→PrL circuits may be one of the mechanisms for these sex differences. Given that aCg layer V express more 5-HT_2A_ receptors than other layers (Santana and Artigas, 2017), an increased number of aCg neurons in layer V projecting to the PrL may be activated by serotonin during stress responses. In such scenario, this would evoke stronger responses in males compared to females. This hypothesis can be supported by interesting evidence: males, contrary to females, can selectively engage PrL cells that project to the dorsal raphe nuclei after exposure to a controllable stress (Baratta et al., 2018). A speculation based on our data is that this engagement of the PrL requires the activation of the aCg→PrL circuit. This activation would be weaker in females due to fewer aCg→PrL projecting neurons, which may explain the sex differences in stress responses.

However, further evidence is needed to make assumptions about the potential sex differences speculated here. First, it is unclear how intra- and intersex variability in the cingulate cortex differs. Second, since the sample size of our data is small, any claims about sex dimorphism in the cingulate cortex need to be supported by additional evidence. Third, although this Perspective article presents a potential hypothesis linking neuroanatomical difference to function, supporting evidence in the literature is sparse. One study has implicated serotonin in the development of social bonding in mandarin voles, but that study focused only on the aCg (Li et al., 2021), emphasizing again the need to use the new neuroanatomical definition of aCg/mCg for rodents in future studies.

## Conclusion

Here we present evidence for a new neuroanatomical definition that divides the mouse cingulate cortex into aCg and mCg rather than Cg1/Cg2. Our tracing data revealed the existence of aCg and mCg neurons projecting to the PrL and their different laminar distribution with sex differences. These findings support the new concept of rodent cingulate cortex proposed in this Perspective article. In future studies of the cingulate cortex, approaching neuroscientific questions from this perspective may shed light on many aspects of cognitive control, emotional regulation, and motivation that lead to behavioral changes. We hope that this perspective will establish the initial foundations and conceptual innovations that will pave the way for future studies focused not only on the cingulate cortex, but also on cortico-cortical circuits.

## Data Availability Statement

The raw data supporting the conclusions of this article will be made available by the authors, without undue reservation.

## Ethics Statement

The animal study was reviewed and approved by the Institutional Animal Care and Use Committees at The University of Alabama at Birmingham.

## Author Contributions

JF-O and MN conceived and designed the project and wrote the first draft of the manuscript. JF-O and OL performed the experiments. JF-O analyzed the data. MN supervised the present study, provided resources, and acquired funding. All authors revised, edited, and approved the manuscript for the final version.

## Conflict of Interest

The authors declare that the research was conducted in the absence of any commercial or financial relationships that could be construed as a potential conflict of interest.

## Publisher’s Note

All claims expressed in this article are solely those of the authors and do not necessarily represent those of their affiliated organizations, or those of the publisher, the editors and the reviewers. Any product that may be evaluated in this article, or claim that may be made by its manufacturer, is not guaranteed or endorsed by the publisher.

## References

[B1] AGordon J. H. Shryne J. E. Southam A. M. (1978). Evidence for a morphological sex difference within the medial preoptic area of the rat brain. *Brain Res.* 148 333–346. 10.1016/0006-8993(78)90723-0 656937

[B2] Ahmed A. KDong K. Sugioka K. Yamadori T. (1995). Afferent projections to the cingulate cortex in albino rats: a study with a retrograde labeling method using fluoro-gold. *Kobe. J. Med. Sci.* 41 247–255. 8869010

[B3] Arnsten A. F. (2009). Stress signalling pathways that impair prefrontal cortex structure and function. *Nat. Rev. Neurosci.* 10 410–422. 10.1038/nrn2648 19455173 PMC2907136

[B4] Avesar D. Stephens E. K. Gulledge A. T. (2018). Serotonergic Regulation of Corticoamygdalar Neurons in the Mouse Prelimbic Cortex. *Front. Neural. Circuits* 12:63. 10.3389/fncir.2018.00063 30131678 PMC6090182

[B5] Baker A. Kalmbach B. Morishima M. Kim J. Juavinett A. Li N. (2018). Specialized Subpopulations of Deep-Layer Pyramidal Neurons in the Neocortex: Bridging Cellular Properties to Functional Consequences. *J. Neurosci.* 38 5441–5455. 10.1523/JNEUROSCI.0150-18.2018 29798890 PMC6001033

[B6] Baratta M. V. Leslie N. R. Fallon I. P. Dolzani S. D. Chun L. E. Tamalunas A. M. (2018). Behavioural and neural sequelae of stressor exposure are not modulated by controllability in females. *Eur. J. Neurosci.* 47 959–967. 10.1111/ejn.13833 29359831 PMC5902414

[B7] Bojak I. Miceli S. Schubert D. A. (2015). gradual depth-dependent change in connectivity features of supragranular pyramidal cells in rat barrel cortex. *Brain Struct. Funct.* 220 1317–1337. 10.1007/s00429-014-0726-8 24569853 PMC4409644

[B8] Brunson K. L. Chen Y. Avishai-Eliner S. Baram T. Z. (2003). Stress and the developing hippocampus: a double-edged sword? *Mol. Neurobiol.* 27 121–136. 10.1385/MN:27:2:121 12777683 PMC3084035

[B9] Chandrasekaran C. Peixoto D. Newsome W. T. Shenoy K. V. (2017). Laminar differences in decision-related neural activity in dorsal premotor cortex. *Nat. Commun.* 8:614. 10.1038/s41467-017-00715-0 28931803 PMC5607001

[B10] Correa-Junior N. D. Renner J. Fuentealba-Villarroel F. Hilbig A. Rasia-Filho A. A. (2020). Dendritic and Spine Heterogeneity of von Economo Neurons in the Human Cingulate Cortex. *Front. Synaptic. Neurosci.* 12:25. 10.3389/fnsyn.2020.00025 32733229 PMC7360805

[B11] Cruz A. M. Kim T. H. Smith R. J. (2021). Monosynaptic Retrograde Tracing From Prelimbic Neuron Subpopulations Projecting to Either Nucleus Accumbens Core or Rostromedial Tegmental Nucleus. *Front. Neural. Circuits* 15:639733. 10.3389/fncir.2021.639733 33732114 PMC7959753

[B12] Dajani D. R. Uddin L. Q. (2015). Demystifying cognitive flexibility: Implications for clinical and developmental neuroscience. *Trends Neurosci.* 38 571–578. 10.1016/j.tins.2015.07.003 26343956 PMC5414037

[B13] de Kock C. P. Bruno R. M. Ramirez A. Meyer H. S. Dercksen V. J. Helmstaedter M. (2012). Cell type-specific three-dimensional structure of thalamocortical circuits in a column of rat vibrissal cortex. *Cereb. Cortex* 22 2375–2391. 10.1093/cercor/bhr317 22089425 PMC3432239

[B14] Etkin A. Egner T. Kalisch R. (2011). Emotional processing in anterior cingulate and medial prefrontal cortex. *Trends Cogn. Sci.* 15 85–93. 10.1016/j.tics.2010.11.004 21167765 PMC3035157

[B15] Fillinger C. Yalcin I. Barrot M. Veinante P. (2017). Afferents to anterior cingulate areas 24a and 24b and midcingulate areas 24a’ and 24b’ in the mouse. *Brain Struct. Funct.* 222 1509–1532. 10.1007/s00429-016-1290-1 27539453

[B16] Franklin K. B. J. Paxinos G. (2013). *The mouse Brain in Stereotaxic Coordinates. Fourth edition. ed.* Amsterdam: Academic Press.

[B17] Gulati S. Cao V. Y. Otte S. (2017). Multi-layer Cortical Ca2+ Imaging in Freely Moving Mice with Prism Probes and Miniaturized Fluorescence Microscopy. *J. Vis. Exp.* 124:e55579. 10.3791/55579 28654056 PMC5608392

[B18] Hoover W. B. Vertes R. P. (2007). Anatomical analysis of afferent projections to the medial prefrontal cortex in the rat. *Brain Struct. Funct.* 212 149–179. 10.1007/s00429-007-0150-4 17717690

[B19] Ibrahim L. A. Schuman B. Bandler R. Rudy B. Fishell G. (2020). Mining the jewels of the cortex’s crowning mystery. *Curr. Opin. Neurobiol.* 63 154–161. 10.1016/j.conb.2020.04.005 32480351 PMC8075042

[B20] JLi X. Chen T. Ren M. Zhuo M. (2009). Characterization of intracortical synaptic connections in the mouse anterior cingulate cortex using dual patch clamp recording. *Mol. Brain* 2:32. 10.1186/1756-6606-2-32 19828050 PMC2770551

[B21] Larkum M. E. Petro L. S. Sachdev R. N. S. Muckli L. A. (2018). Perspective on Cortical Layering and Layer-Spanning Neuronal Elements. *Front. Neuroanat.* 12:56. 10.3389/fnana.2018.00056 30065634 PMC6056619

[B22] Laubach M. Amarante L. M. Swanson K. White S. R. (2018). What, If Anything, Is Rodent Prefrontal Cortex? *eNeuro* 5:ENEURO.315–ENEURO.318. 10.1523/ENEURO.0315-18.2018 30406193 PMC6220587

[B23] Li L. Zhang L. Z. He Z. X. Ma H. Zhang Y. T. Xun Y. F. (2021). Dorsal raphe nucleus to anterior cingulate cortex 5-HTergic neural circuit modulates consolation and sociability. *Elife* 10:e67638. 10.7554/eLife.67638 34080539 PMC8213405

[B24] Liu C. Y. Lai W. S. (2021). Functional neuroanatomy and neural oscillations during social eavesdropping in male golden hamsters. *Horm. Behav.* 127:104881. 10.1016/j.yhbeh.2020.104881 33127368

[B25] McEwen B. S. Milner T. A. (2017). Understanding the broad influence of sex hormones and sex differences in the brain. *J. Neurosci. Res.* 95 24–39. 10.1002/jnr.23809 27870427 PMC5120618

[B26] Qadir H. Krimmel S. R. Mu C. Poulopoulos A. Seminowicz D. A. Mathur B. N. (2018). Structural Connectivity of the Anterior Cingulate Cortex, Claustrum, and the Anterior Insula of the Mouse. *Front. Neuroanat.* 12:100. 10.3389/fnana.2018.00100 30534060 PMC6276828

[B27] Robbins T. W. (2005). Controlling stress: how the brain protects itself from depression. *Nat. Neurosci.* 8 261–262. 10.1038/nn0305-261 15746909

[B28] Sandi C. Haller J. (2015). Stress and the social brain: behavioural effects and neurobiological mechanisms. *Nat. Rev. Neurosci.* 16 290–304. 10.1038/nrn3918 25891510

[B29] Santana N. Artigas F. (2017). Laminar and Cellular Distribution of Monoamine Receptors in Rat Medial Prefrontal Cortex. *Front. Neuroanat.* 11:87. 10.3389/fnana.2017.00087 29033796 PMC5625028

[B30] Shackman A. JSalomons T. V. Slagter H. A. Fox A. S. Winter J. J. Davidson R. J. (2011). The integration of negative affect, pain and cognitive control in the cingulate cortex. *Nat. Rev. Neurosci.* 12 154–167. 10.1038/nrn2994 21331082 PMC3044650

[B31] Spratling M. W. (2002). Cortical region interactions and the functional role of apical dendrites. *Behav. Cogn. Neurosci. Rev.* 1 219–228. 10.1177/1534582302001003003 17715594

[B32] Stuart G. J. Spruston N. (2015). Dendritic integration: 60 years of progress. *Nat. Neurosci.* 18 1713–1721. 10.1038/nn.4157 26605882

[B33] Uddin L. Q. Yeo B. T. T. Spreng R. N. (2019). Towards a Universal Taxonomy of Macro-scale Functional Human Brain Networks. *Brain Topogr.* 32 926–942. 10.1007/s10548-019-00744-6 31707621 PMC7325607

[B34] van Heukelum S. Mogavero F. van de Wal M. A. E. Geers F. E. França A. S. C. Buitelaar J. K. (2019b). Gradient of Parvalbumin- and Somatostatin-Expressing Interneurons Across Cingulate Cortex Is Differentially Linked to Aggression and Sociability in BALB/cJ Mice. *Front. Psychiatry* 10:809. 10.3389/fpsyt.2019.00809 31803076 PMC6873752

[B35] van Heukelum S. Drost L. Mogavero F. Jager A. Havenith M. N. Glennon J. C. (2019a). Aggression in BALB/cJ mice is differentially predicted by the volumes of anterior and midcingulate cortex. *Brain Struct. Funct.* 224 1009–1019. 10.1007/s00429-018-1816-9 30560374 PMC6499875

[B36] van Heukelum S. Mars R. B. Guthrie M. Buitelaar J. K. Beckmann C. F. Tiesinga P. H. E. (2020). Where is Cingulate Cortex? A Cross-Species View. *Trends Neurosci.* 43 285–299. 10.1016/j.tins.2020.03.007 32353333

[B37] Vogt B. A. (2016). Midcingulate cortex: Structure, connections, homologies, functions and diseases. *J. Chem. Neuroanat.* 74 28–46. 10.1016/j.jchemneu.2016.01.010 26993424

[B38] Vogt B. A. Finch D. M. Olson C. R. (1992). Functional heterogeneity in cingulate cortex: the anterior executive and posterior evaluative regions. *Cereb Cortex* 2 435–443. 10.1093/cercor/2.6.435-a 1477524

[B39] Vogt B. A. Paxinos G. (2014). Cytoarchitecture of mouse and rat cingulate cortex with human homologies. *Brain Struct. Funct.* 219 185–192. 10.1007/s00429-012-0493-3 23229151

[B40] Witelson S. F. Glezer I. I. Kigar D. L. (1995). Women have greater density of neurons in posterior temporal cortex. *J. Neurosci.* 15 3418–3428. 10.1523/JNEUROSCI.15-05-03418.1995 7751921 PMC6578233

